# Impact of Pretreatment Degree and Enzyme Type on the Production of Radical Scavenging and Antiproliferative Peptides from Starfish

**DOI:** 10.3390/md24030120

**Published:** 2026-03-23

**Authors:** Naveen Kumar Vate, Elahe Sharifi, Alessandro Coppola, Eleonora Montuori, Ingrid Undeland, Donatella de Pascale, Daniela Coppola, Mehdi Abdollahi

**Affiliations:** 1 Department of Life Sciences–Food and Nutrition Science, Chalmers University of Technology, 412 96 Gothenburg, Sweden; elahes@chalmers.se (E.S.); undeland@chalmers.se (I.U.); 2 Department of Fish Processing Technology, School of Fisheries, Centurion University of Technology and Management, Paralakhemundi 761221, Odisha, India; 3 Department of Ecosustainable Marine Biotechnology, Stazione Zoologica Anton Dohrn, Via Ammiraglio Acton, 55, 80133 Naples, Italy; alessandro.coppola@szn.it (A.C.); eleonora.montuori@szn.it (E.M.); donatella.depascale@szn.it (D.d.P.); daniela.coppola@szn.it (D.C.)

**Keywords:** starfish, *Asterias rubens*, protein hydrolysate, radical scavenging activity, antiproliferative, collagen

## Abstract

Enzymatic hydrolysis is one of the effective methods used to obtain the bioactive peptides from marine resources. This study aimed to evaluate effect of the enzyme type (Food Pro PNL (FP), Corolase8000 (C8), and Corolase7089 (C7)) and biomass pretreatment level (whole starfish (SF), deproteinized (DPSF) as well as deproteinized and demineralized starfish (DPDMSF)) on the hydrolysate yield, degree of hydrolysis (DH), generated peptides’ molecular weight (MW), and in vitro radical scavenging and antiproliferative effects. Regardless of the enzyme used, deproteinization reduced the hydrolysate yield (<8% *dw*/*ww*) and DH (<5%), but also adding demineralization, in combination with C8, resulted in an equal yield (15%) and DH (>40%) to SF. However, the protein content of hydrolysates from DPSF and DPDMSF was higher than that prepared from SF. C8 was not effective in hydrolyzing SF but was the only effective enzyme in hydrolyzing DPDMSF. The peptides’ MW distribution strongly depended on the pretreatment and enzyme type, mostly ranging from 17 to 70 kDa. Glycine content was higher in hydrolysates from DPSF and DMDPSF, indicating their collagenous nature. Hydrolysates from DPSF, rich in collagenous peptides, showed medium MW but the highest radical scavenging activity. Only SF-FP hydrolysate, rich in non-collagenous peptides, showed antiproliferative activity against melanoma cancer cells. Overall, the findings demonstrate that upstream biomass pretreatment and enzyme selection directly govern the yield and bioactivity of starfish protein hydrolysates, providing a rational basis for designing starfish protein hydrolysates with targeted functional properties.

## 1. Introduction

Protein hydrolysates are currently produced commercially from various sources, including plant, dairy, chicken, fish, and shellfish. Apart from having many functional properties, protein hydrolysates have been shown to exhibit bioactivities such as antihypertensive, antagonist, immunomodulatory, antithrombotic, antioxidant, anticancer, and antimicrobial activities [1,2,3]. In addition to fish and its by-products, by-catches have been exploited to obtain bioactive peptides. Marine resources such as sea cucumbers, jellyfish [4,5,6], marine echiuroid worm [7], blue mussel [8], and ark shell [9] have been utilized for the production of protein hydrolysates, showing angiotensin-I converting enzyme (ACE) inhibitory, anti-hypertensive effects and anti-coagulant activity.

Marine-derived protein hydrolysates have attracted increasing attention as sources of multifunctional bioactive peptides. Numerous studies have reported that peptides derived from fish, mollusks, and other marine organisms possess antioxidant properties and can inhibit oxidative damage through radical scavenging and metal-chelating mechanisms [10]. In addition, several marine peptides have demonstrated antiproliferative or anticancer activities through mechanisms such as induction of apoptosis, modulation of cell-cycle progression, and interference with cellular signaling pathways [11]. Despite these advances, limited research has explored how biomass composition and enzymatic hydrolysis strategies influence the generation of peptides with distinct bioactivities from underutilized marine resources such as starfish.

The starfish *Asterias pectinifera*, known to cause problems in the aquaculture industry, has been identified as a source of low-molecular-weight (LMW) collagen peptides, which promote wound healing, bone regeneration, and skin protection [12]. Moreover, collagen peptides produced from *A. pectinifera* have been used for encapsulating elastic nanoliposomes for cosmetic applications [12]. The common starfish *Asterias rubens* is an invasive species creating problems in mussel farming. It is harvested as a by-catch along with the mussel and thrown away as waste. It has been reported that the starfish *A. rubens* and *Marthasterias glacialis* are also predators of scallops and can cause losses of up to 11% of young scallops in sea farming [13]. Moreover, the common starfish *A. rubens* has successfully been utilized as a source of collagen in our previous study [14], but to our knowledge, has not been targeted for the production of protein hydrolysates comprising bioactive peptides.

The enzymatic hydrolysis process for producing bioactive peptides from starfish can target either the entire pool of starfish proteins or its collagenous tissues. Utilizing the entire starfish biomass ensures more efficient resource use but generates peptides comprising both non-collagenous and, to a lesser extent, collagenous peptides. Conversely, producing exclusively collagenous peptides requires sequential pretreatments, such as deproteinization to remove non-collagenous proteins and/or demineralization. While these additional steps increase the cost and reduce the sustainability of the process, they may yield distinct collagenous peptides with unique bioactivities and applications. We speculate that the efficacy of various proteases in hydrolyzing the biomass is impacted by variations in the biomass composition, particularly the ratio of non-collagenous to collagenous proteins and the mineral content. This would not only impact the process viability but also influence the molecular weight (MW) and bioactivity of the resulting peptides. To date, the effect of the biomass pretreatment degree and enzyme type (based on targeted protein substrates) on the hydrolysis efficiency and peptide characteristics of starfish remains unexplored.

The present study aimed to evaluate the effect of three different biomass pretreatment levels—no pretreatment (i.e., entire starfish), deproteinized starfish, and deproteinized and demineralized starfish, as well as the type of enzyme applied, including Food Pro^®^ PNL, Corolase^®^ 8000, and Corolase^®^ 7089. The differences in specificity, mechanisms of action, and substrate broadness of these three enzymes [15] were hypothesized to affect the hydrolysate yield as well as the degree of hydrolysis, MW distribution, amino acid composition, and bioactivity of the generated peptides. The latter mainly comprised the antioxidant and antiproliferative activities.

## 2. Results and Discussion

### 2.1. Yield and Crude Protein Content

**Figure 1:**
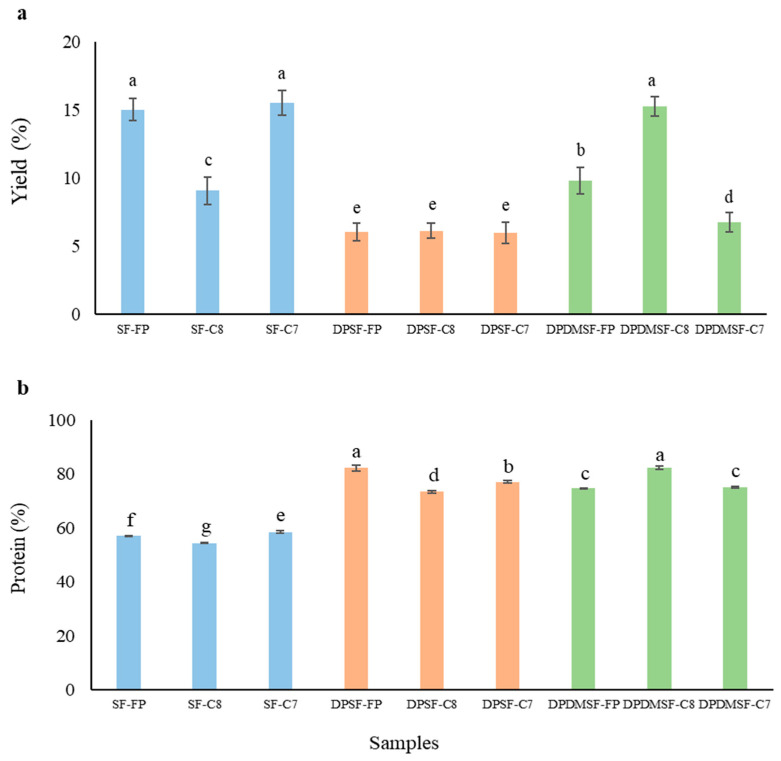
Weight yield (*dw*/*ww*) (**a**) and crude protein content (**b**) of hydrolysates produced from whole starfish (SF), deproteinized starfish (DPSF), as well as deproteinized and demineralized starfish (DPDMSF) by Food Pro PNL (FP), Corolase 8000 (C8), or Corolase 7089 (C7). Data show mean values ± SD (n = 3). Different small letters show significant differences (*p* < 0.05).

The yields of protein hydrolysates obtained from the starfish biomasses after different pretreatments using different enzymes are depicted in Figure 1a. The hydrolysates SF-FP, SF-C7, and DPDMSF-C8 showed the highest yield (~15% *dw*/*ww*), while all the DPSF hydrolysates (obtained using all three enzymes) showed the lowest yield (<7% *w*/*w*). The results indicated that enzyme activity varies depending on the raw material purity and enzyme type. In particular, the enzymes Food Pro PNL and Corolase 7089 showed good activity on SF, likely due to the availability of both collagenous and non-collagenous proteins. In contrast, Corolase 8000, which is mainly used for animal protein hydrolysis, exhibited greater activity on DPDMSF, suggesting it has higher specificity for collagenous proteins and is more effective in the absence of minerals or non-collagenous proteins. Minerals can interfere with enzyme–substrate interactions, potentially inhibiting enzyme activity [16]. On the other hand, the DPDMSF sample, consisting almost only of pure collagenous protein, resulted in a higher yield compared to DPSF, indicating the importance of substrate availability and protein purity for maximizing enzymatic activity.

The differences in yield observed between the same raw materials treated with different enzymes highlight the role of enzyme specificity and substrate affinity. Corolase 8000 showed a superior performance on DPDMSF, suggesting that it is highly effective in hydrolyzing starfish collagen but sensitive to impurities such as minerals, which may hinder its activity [16]. These findings underscore the importance of selecting appropriate pretreatment methods and enzyme types to optimize the hydrolysate yield. For example, the highest yield of pepsin-soluble collagen peptides was obtained from the skin of sturgeon (Acipenser baerii × Huso huso) by using trypsin when compared to pepsin, papain, alcalase, and flavorzyme [17]. Nurilmala et al. [18], reported a yield of 22.79% (*dw*/*ww*) for collagen peptides from yellowfin tuna skin when acetic acid and papain were used for extraction.

The crude protein content in the hydrolysates (Figure 1b), which represents the purity of the peptides, was highest in DPSF-FP (81%) and DPDMSF-C8 (80%), followed by DPSF-C7 (75%); on the contrary, it was least in SF-C8 (54%) (Figure 1b). The hydrolysates prepared from SF using all three enzymes had the lowest crude protein content, indicating that with the higher yield, the protein purity went down. This is more likely due to the presence of minerals found mainly in the calcareous endoskeleton of starfish, which has partly dissolved in the hydrolysate and remained during the freeze-drying step [16]. However, we assume that during the deproteinization step, most of the easily extractable minerals such as calcium and phosphorus were already removed together with the non-collagenous proteins, explaining why the crude protein content was as high in DPSF- as in DPDMSF-derived hydrolysates. The latter likely contained mostly collagen peptides, explaining their significantly (*p* < 0.05) higher purity than SF-derived hydrolysates. Hydrolysates produced from salmon (*Salmo salar*) skin using the serine endopeptidase alcalase had a protein content as high as 89.53 ± 0.51% [19], while hydrolyzed cod by-products had a crude protein content of around 80%, similar to that in DPSF- and DPDMSF-hydrolysates [20].

### 2.2. Degree of Hydrolysis

**Figure 2:**
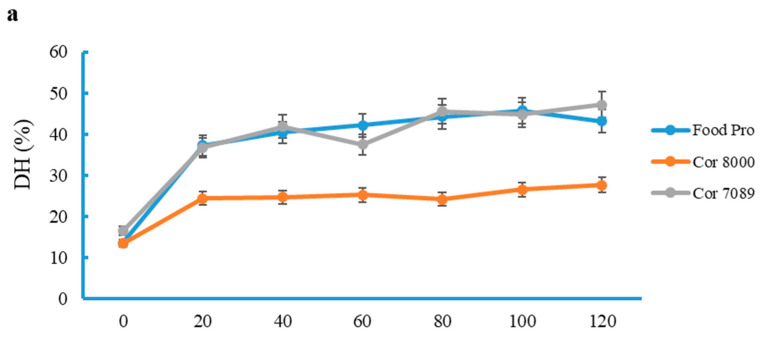

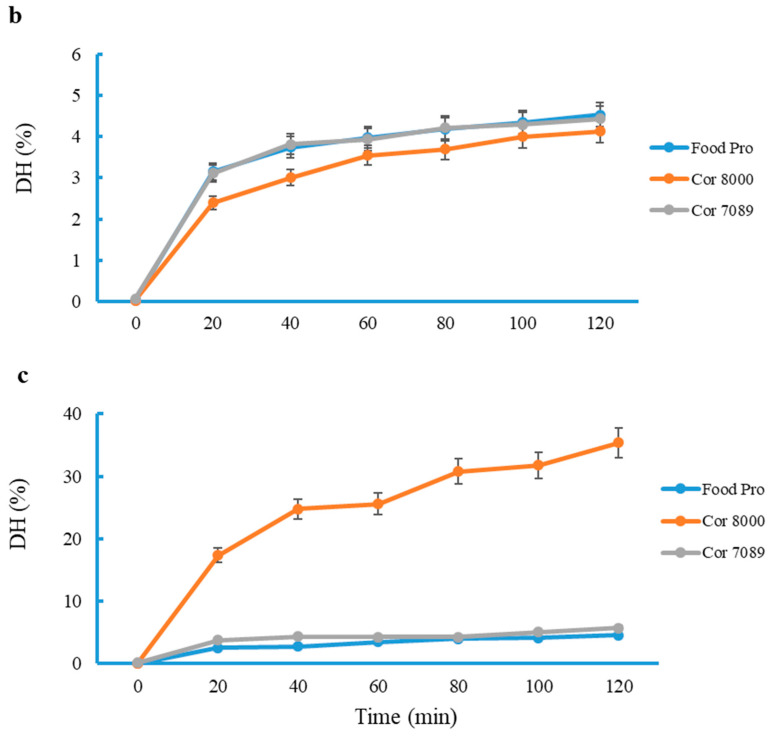
Degree of hydrolysis (DH) obtained from different enzymes when applied on whole starfish (SF) (**a**), deproteinized starfish (DPSF) (**b**), as well as deproteinized and demineralized starfish (DPDMSF) (**c**) during the hydrolysis process for two hours. Data show mean values ± SD (n = 3).

The degree of hydrolysis (DH) obtained over time from SF, DPSF, and DPDMSF using different enzymes during hydrolysis is depicted in Figure 2.

The Food Pro PNL and Corolase 7089 showed significantly higher DHs for SF compared to Corolase 8000, with around 40% after 20 min of hydrolysis. However, Corolase 8000 only reached a DH of 25% for this biomass in the same period, which remained constant until the end of the process. Almost the same trend was seen for DPSF, but the achieved DH was 10-fold lower compared with SF hydrolysate; the maximum DH was only 4.6% after 100 min of hydrolysis. Food Pro PNL and Corolase 7089 resulted in almost the same DH (<5%) for the DPDMSF after 120 min. On the contrary, Corolase 8000 was very effective on the DPDMSF, resulting in a DH value of ~30% after 80 min. This was in agreement with the yield result (Figure 1a). Overall, the DH for DPSF was the lowest, which was likely due to the removal of non-collagenous proteins and the presence of a small amount of minerals, which affected the enzyme activity. Hydrolysates prepared from sea cucumber viscera, urchin digestive tract, and urchin gonads using alcalase had DHs of 5.6%, 4.6% and 7.0% respectively, which were much lower compared to other studies involving fish by-products as raw materials [21]. The higher DH of SF than that of DPSF was likely due to the enzyme acting on both collagenous and non-collagenous proteins. The Corolase 8000 enzyme showed a higher activity for DPDMSF containing only collagenous proteins than for SF and DPSF, indicating its suitability for preparing collagen hydrolysates or collagen peptides. Overall, collagen is a highly durable protein with a naturally evolved tropo-helical structure that is difficult to hydrolyze [22], which was proven while using Food Pro PNL and Corolase 7089. The lower activity of Corolase 8000 towards SF and DPSF compared to DPDMSF indicated that its activity was affected both by non-collagenous proteins and by the presence of minerals. As stated above, the sorption of enzymes on minerals likely decreases the enzyme activity due to the hindrance of active sites [23]. Overall, the results indicated that the enzymes Food Pro PNL and Corolase 7089 could be suitable for the preparation of hydrolysates from non-collagenous starfish proteins, and Corolase 8000 for the preparation of collagenous hydrolysates.

### 2.3. Amino Acid Composition of Hydrolysates

The amino acid composition of the hydrolysates obtained from starfish using different enzymes is shown in Table 1.

Glycine was found in the highest quantity in all the hydrolysates, regardless of pretreatment and enzyme type. Hydrolysates prepared from SF had a lower glycine content compared to those prepared from DPSF and DPDMSF, since they contained both collagenous and non-collagenous proteins, while the latter two had mainly collagenous proteins. Among all samples, the highest glycine content was observed in the hydrolysate prepared from DPSF using Corolase 8000, followed by that prepared from DPDMSF using the same enzyme. This was likely due to the higher collagenous protein content in DPSF and DPDMSF. The amino acid composition of hydrolysates from DPDMSF was comparable to the distinctive amino acid composition for collagen, in which glycine is found at every third amino acid residue [24]. The hydrolysate prepared from SF using Corolase 8000 had the lowest glycine content, supporting its suitability for collagenous proteins. The hydrolysates prepared from SF had glutamine and asparagine as the second and third major amino acids, which is in agreement with the amino acid content reported for the starfish *Acanthaster planci* [25]. The hydrolysates prepared from DPDMSF had glutamine and proline as the second and third major amino acids, suggesting that these were collagen peptides, as collagen subunits constitute a repeating chain of Glycine-X-Y, where X and Y vary, but are usually occupied by proline and hydroxyproline, respectively [26]. The amino acid composition of hydrolyzed DPSF and DPDMSF was comparable with that of collagen peptides from *A. pectinifera* [12] as well as collagen extracted from *A. planci* [27], seastar *A. amurensis* [28], and purple sea urchin (*Anthocidaris crassispina*) [29], all with glycine as the main amino acid.

### 2.4. Molecular Weight Distribution of the Starfish Hydrolysates

**Figure 3:**
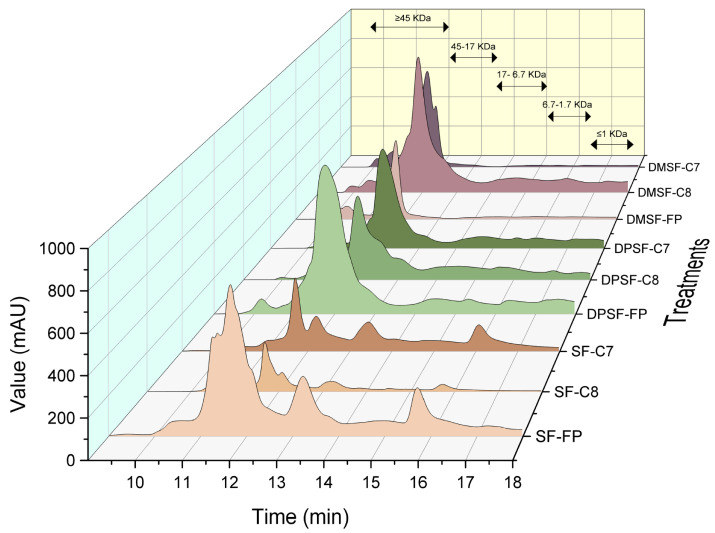
Chromatogram of starfish protein hydrolysates prepared using different enzymes, 1: SF-FP (Whole Starfish with Food Pro PNL); 2: SF-C8 (Whole Starfish with Corolase 8000); 3: SF-C7 (Whole Starfish with Corolase 7089); 4: DPSF-FP (Deproteinized Starfish with Food Pro PNL); 5: DPSF-C8 (Deproteinized Starfish with Coroalse 8000); 6: DPSF-C7 (Deproteinized Starfish with Corolase 7089); 7: DPDMSF-FP (Demineralized Starfish with Food Pro PNL); 8: DPDMSF-C8 (Demineralized Starfish with Corolase 8000); 9: DPDMSF-C7 (Demineralized Starfish with Corolase 7089).

The molecular weight distribution of the starfish protein hydrolysates was evaluated by size exclusion chromatography (SEC) (Figure 3). Across all treatments, a dominant peak was observed in the 17–60 kDa range, with the apex typically around 45 kDa. Peak height, breadth, and the presence of secondary peaks varied markedly with both the pretreatment and enzyme type.

Hydrolysates from whole starfish (SF; chromatograms 1–3) exhibited the broadest molecular weight profiles. In addition to the main 45 kDa peak, distinct secondary peaks were detected near 17 kDa and 6.7 kDa. These lower-molecular-weight peaks were most pronounced in samples hydrolyzed with Food Pro PNL and Corolase 7089, whereas Corolase 8000 produced a narrower distribution. The broader profiles for SF hydrolysates likely reflect the more diverse protein substrate pool, including myofibrillar, sarcoplasmic, and collagenous proteins, enabling multiple cleavage patterns and a wider peptide size range. These SEC observations are consistent with the DH data, where SF-FP and SF-C7 hydrolysates showed a higher DH than SF-C8.

Deproteinized starfish (DPSF; chromatograms 4–6) showed markedly narrower chromatographic profiles dominated by the 17–45 kDa region, with minimal signals below 6.7 kDa. Since deproteinization leaves mainly collagenous proteins, which are structurally more resistant to enzymatic cleavage, these samples showed limited hydrolysis regardless of enzyme type. The high mineral content may also have reduced enzyme accessibility by sterically hindering cleavage sites. In addition to steric hindrance, the high mineral fraction, primarily calcium carbonate typical of starfish ossicles, may stabilize collagen fibrils through mineral–protein interactions, limiting enzyme penetration and reducing the number of accessible cleavage sites. Such mineral–collagen associations can therefore restrict hydrolysis efficiency and contribute to the persistence of higher-molecular-weight peptide fractions.

The most intensively pretreated material, deproteinized and demineralized starfish (DPDMSF; chromatograms 7–9), produced enzyme-dependent MW profiles. In general, the removal of minerals likely disrupted these mineral–protein associations, increasing the exposure of collagen triple helices and facilitating enzymatic access, which in turn promoted more extensive peptide fragmentation. Notably, only Corolase 8000 yielded a markedly broader distribution with a substantial fraction of peptides < 6.7 kDa, indicating extensive collagen breakdown. This can be explained by the composition of Corolase 8000 enzyme, which contains both endopeptidase and exopeptidase activities, enabling sequential cleavage, first at accessible internal sites, followed by progressive trimming from peptide termini. Such dual action is particularly important for collagen-rich substrates, which offer limited cleavage opportunities for endopeptidases alone. In contrast, Food Pro PNL, predominantly an endopeptidase, generated larger peptides, while Corolase 7089, although effective in mixed-protein substrates, appears less suited for collagen-only substrates. The removal of minerals in DPDMSF likely enhanced this effect by eliminating protein–mineral complexes and increasing enzyme access to collagen fibrils. The DH results support this interpretation, as only DPDMSF–C8 achieved DH values exceeding 5%.

These findings are consistent with prior work showing that efficient hydrolysis of collagen requires both substrate accessibility and enzyme systems with complementary activities. Hou and Chen [9], for example, demonstrated that endo- and exopeptidase mixtures (e.g., flavorzyme) and aspartic endopeptidases (e.g., pepsin) were more effective at producing low-molecular-weight collagen peptides than serine proteases such as papain, alcalase, or trypsin. In the present study, the combination of extensive pretreatment and Corolase 8000’s broad hydrolytic mode allowed for maximal peptide size reduction in collagen-rich starfish biomass.

### 2.5. Radical Scavenging Activity

**Figure 4:**
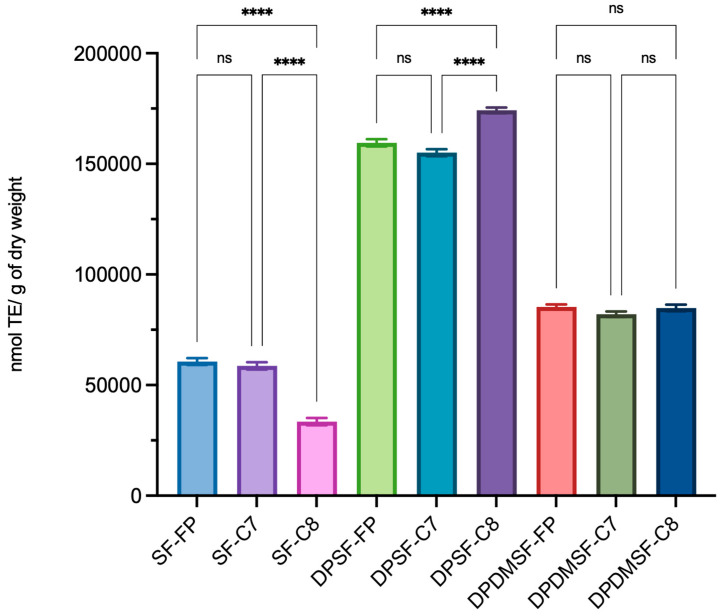
ABTS radical scavenging activity of whole starfish (SF), deproteinized starfish (DPSF), and deproteinized and demineralized starfish (DPDMSF) hydrolysates, obtained by Food Pro PNL (FP), Corolase 8000 (C8), or Corolase 7089 (C7). Data are the mean values of three independent experiments performed in three technical replicates. The antioxidant activity is expressed as nmol of TE per mg/g of dry weight ± standard deviation. ANOVA ordinary one-way Bonferroni’s multiple comparison 0.1234 (ns), <0.0001 (****).

In the present study, starfish hydrolysates were analyzed for their radical scavenging activity by the ABTS assay. This spectrophotometric method is extensively used as a screening assay for determining the antioxidant activity of natural products [30]. The ABTS radical scavenging activity is shown in Figure 4. Results highlight that the radical scavenging activity of DPSF hydrolysates was significantly higher compared with those from SF or DPDMSF. Moreover, DPSF hydrolysates showed higher antioxidant activity irrespective of the enzymes used for the hydrolysis. However, DPSF-C8 showed the best ABTS radical scavenging activity (174,200 nmol TE/g dw). The DPDMSF hydrolysates showed a radical scavenging activity that was approximately half of that of DPSF hydrolysates (DPDMSF-FP, DPDMSF-C7, and DPDMSF-C8 with values of 85,300, 82,000, and 84,800 nmol TE/g dw, respectively). Lower radical scavenging activity was observed for SF hydrolysates.

The higher ABTS radical scavenging activity in the DPSF samples compared to those from SF could be explained by a higher percentage of collagen peptides capable of scavenging free radicals. However, although the subsequent demineralization process results in a product with a higher concentration of pure collagen peptides, the removal of minerals likely compromises the structure and biological activity of the peptides. This could explain the lower ABTS radical scavenging activity of collagen peptides in DPDMSF hydrolysate. Similarly, it has previously been shown that collagen peptides (<3 kDa) derived from bovine bones, obtained through enzymatic hydrolysis using bromelain, cathepsin B, and collagenase B, showed significant antioxidant activity in the presence of minerals such as calcium, magnesium, and phosphorous [31]. These peptides were effective in scavenging various free radicals, reducing oxidizing agents, and preventing lipid peroxidation; it was shown that the antioxidant activity of peptides depended significantly on both the type of enzyme used and the assay performed to evaluate their antioxidant potential. Although bromelain and cathepsin B (collagen-nonspecific enzymes) were capable of producing antioxidant peptides, the most potent antioxidant effects in all tested assays were observed in peptides obtained by collagenase B. These results therefore underline the importance of enzymatic specificity in the production of functional peptides, suggesting that collagenase B might be particularly effective for peptides with strong antioxidant functions. In addition, the antioxidant activity was further increased by mixing peptides produced by collagenase B with peptides obtained from bromelain and cathepsin B, suggesting a synergistic interaction between the resulting peptides. These results highlight the potential benefits of using a multienzyme approach in the production of functional ingredients for nutraceutical applications [31].

In the case of freshwater carp (*Catla catla*), it was observed that the fish protein hydrolysates prepared using the cysteine endopeptidase bromelain had higher free radical-scavenging activity compared to those prepared using other proteases, such as alcalase, flavorzyme, and protamex, suggesting, also in this case, an enzyme-specific influence [32].

### 2.6. In Vitro Antiproliferative Test

To explore potential bioactivities, the starfish hydrolysates were tested for their possible cytotoxic and antiproliferative activity against human malignant melanoma cells A2058 and normal fibroblast MRC-5 by using the MTT test. The experiment was designed as a preliminary screening to determine whether these compounds exert a biological effect that could justify further investigation.

**Figure 5:**
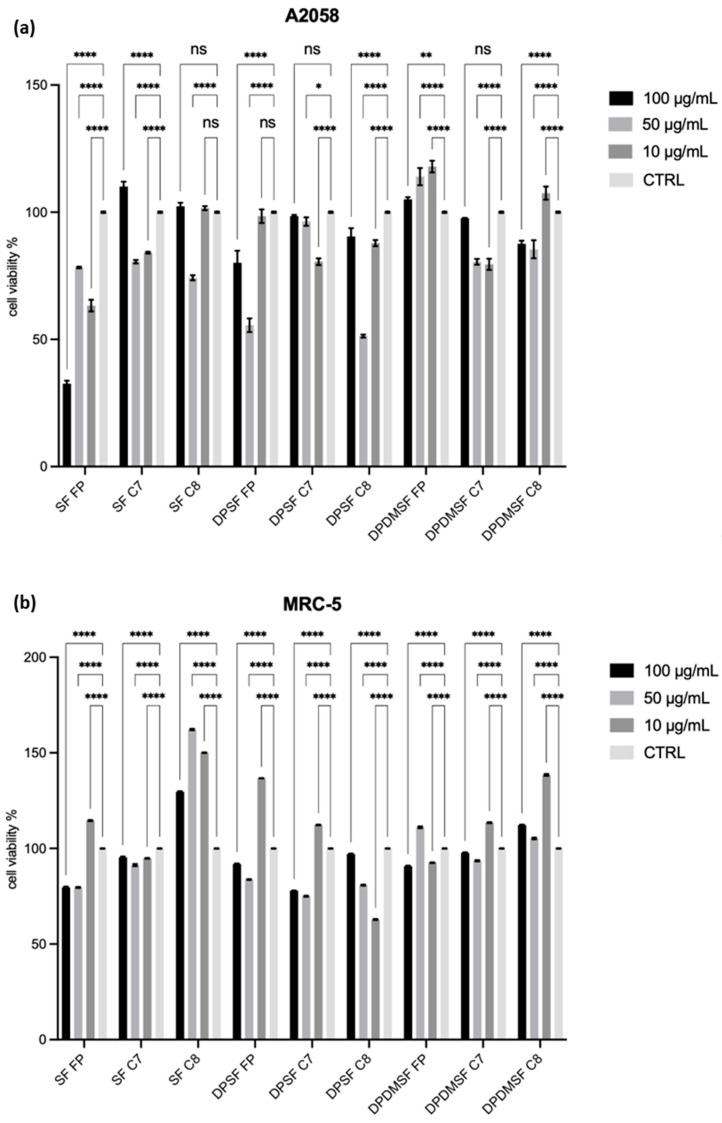
In vitro antiproliferative effect of whole starfish (SF), deproteinized starfish (DPSF), and deproteinized and demineralized starfish (DPDMSF) hydrolysates, obtained by Food Pro PNL (FP), Corolase 8000 (C8), or Corolase 7089 (C7) on A2058 cell line (malignant melanoma) (**a**) and MRC-5 (control cell line) (**b**) and the respective controls only with the culture medium. Results are expressed as percentage of cell survival after 72 h exposure (n = 3). ANOVA ordinary one-way Dunnett’s multiple comparison 0.1234 (ns), 0.0332 (*), 0.0021 (**), <0.0001 (****).

The two cell lines were incubated in the presence of three concentrations for each sample (10 µg/mL, 50 µg/mL, and 100 µg/mL) and in their absence. After 72 h (Figure 5) of incubation at 37 °C with the samples, cell survival was measured by the MTT test. The screening was performed in triplicate. As shown in Figure 5b, at the end of the 72 h treatment, following a comparison with the results obtained under the same experimental conditions on MRC-5 control cells, SF-FP hydrolysate showed an antiproliferative activity against A2058 melanoma cells. In particular, SF-FP showed a reduction in cell proliferation A2058 to 33% at 100 µg/mL, 78% at 50 µg/mL and 68% at 10 µg/mL. In contrast, MRC-5 fibroblast maintains a significantly higher viability (80% at 100 µg/mL, 80% at 50 µg/mL, and 115% at 10 µg/mL); this differential response suggests a preliminary selectivity of SF-FP toward tumor cells, rather than nonspecific cytotoxicity. The limited impact on MRC-5 cells supports the hypothesis that the SF-FP sample may specifically target mechanisms related to melanoma cell growth and survival. Moreover, this behavior could be due to intrinsic differences between malignant and normal cells, such as membrane composition, metabolic rate, redox balance, or activation of stress-related signaling pathways, which could render melanoma cells more susceptible to specific bioactive peptides present in SF-FP.

All the other hydrolysates, even if they decreased cell viability, did not show a significant reduction below 50%, such as DPSF-FP, which reduced cell viability to 56% in A2058 at 50 µg/mL and 80% at 100 µg/mL, and DPSF-C8, which showed a reduction in viability to 52% at 50 µg/mL. This could be due to the presence of non-collagenous peptides with potential anticancer activities in SF-FP. These peptides could directly interact with proteins involved in the growth and survival of cancer cells, or with the membranes of melanoma cells, altering their integrity and inducing apoptosis [33]. Additionally, SF-FP could influence the regulation of the p38 signaling pathway, thereby activating pro-apoptotic factors. It is also important to consider that SF-FP may consist of a combination of peptides, suggesting the possibility of a synergistic effect that enhances the antiproliferative activity on tumor cells.

There is some previous evidence in the literature on the cytotoxic and antiproliferative effects of compounds extracted from various starfish species on melanoma cell lines. For example, a study by Lee et al. [34] focused on extracting a proteic toxin known as CAV (identified as a plancitoxin I protein), which is derived from the venom of the starfish *Acanthaster planci*. This protein showed antiproliferative activity against the malignant melanoma cell line A375.S2, effectively inducing apoptotic events. The mechanism behind this activity was linked to the interaction with the mitochondrial membrane and the regulation of the p38 signaling pathway. Likewise, new triterpene glycosides pacificusosides D–K extracted from the starfish *Solaster pacificus* showed high selective antiproliferative activity against the SK-MEL-2 melanoma cell line [35]. Our findings are also in line with the outcome of a recent review that highlights that starfish are sources of high-value natural compounds, including peptides, which exhibit a significant anticancer effect against different human cancer cell lines [36]. Among mechanisms for these effects are the regulation of cell cycle mediators and the apoptosis gene.

## 3. Materials and Methods

### 3.1. Raw Materials

Fresh common starfish (*A. rubens*) were collected from local mussel farmers in the outskirts of Orust, Sweden. The starfish were covered with ice and transported to the lab. They were washed with cold water upon arrival in the lab, packed, and stored at −80 °C until further use.

### 3.2. Chemicals

All chemicals and reagents were of analytical grade. The enzyme Food Pro^®^ PNL was obtained from IFF Bioscience (New York, NY, USA), and Corolase^®^ 7089 and Corolase^®^ 8000 were obtained AB Enzymes GmbH, Darmstadt, Germany. Food Pro PNL (“FP”) is a microbially produced neutral protease. Corolase 7089 (“C7”) is a liquid-formulated, bacterial protease, which hydrolyzes a broad range of substrates, at neutral pH. Corolase 8000 (“C8”) is a fungal alkaline protease ideal for hydrolyzing proteins under neutral conditions. All three enzymes are classified as endopeptidases, and they have been described as efficient in producing collagen peptides, but also for hydrolyzing animal protein, such as from fish and meat, in a broader sense (Food Pro PNL is applied for preparation of collagen peptides, meat extracts, etc., Corolase 7089 is used for the hydrolysis of proteins from several animal and plant sources, and Corolase 8000 is primarily applied for animal protein hydrolysis). EDTA, tris (hydroxymethyl) aminomethane, sodium dodecyl sulphate (SDS), β-mercaptoethanol (β-ME), glycerol, bovine serum albumin, and potassium persulfate (K_2_S_2_O_8_) (dipotassium peroxdisulfate) were purchased from Sigma-Aldrich Corp. (St. Louis, MO, USA). Sodium hydroxide, hydrochloric acid, and sodium chloride were procured by Scharlo (Scharlo Co., Barcelona, Spain). Trolox (6-hydroxy-2,5,7,8-tetramethylchroman-2-carboxylic acid) and ABTS [2,20-Azinobis (3-ethylben-zothiazoline-6-sulfonic acid) diammonium salt] were obtained from Santa-Cruz Biotechnology (Santa Cruz, CA, USA).

### 3.3. Preparation of Protein Hydrolysates

#### 3.3.1. Biomass Pretreatment

The frozen starfish were thawed in tight plastic bags under cold tap water. The samples were then chopped into small pieces (0.5 mm × 0.5 mm) before being used for hydrolysis. A part of the sample was subjected to deproteinization by mixing the chopped sample with 0.1 M NaOH in a 1:10 ratio (*w*/*v*) and subjecting it to homogenization at 4000 rpm for 2.5 min with a Silverson homogenizer (L5M, Silverson, East Longmeadow, MA, USA). The homogenized sample was centrifuged at 2000× *g* for 2 min at 4 °C, and the supernatant was discarded. The resulting precipitate was mixed with cold water in a 1:10 ratio (*w*/*v*), and the pH was adjusted to 7.4. Then, it was dewatered by centrifuging at 5000× *g* for 5 min. The precipitate was then subjected to enzymatic hydrolysis. For demineralization, the deproteinized sample was mixed with 0.5 M EDTA-2Na solution, keeping the ratio of 1:15 (*w*/*v* sample to solution). The demineralization was carried out in a cold room (4 °C) for 24 h under stirring. The EDTA solution was exchanged for a fresh solution at 12 h. Residual EDTA was removed from demineralized samples by washing with cold water. Then, dewatering of the samples was achieved by centrifugation at 5000× *g* (5 min at 4 °C).

Thus, the 3 samples emerged, namely chopped whole starfish (SF), deproteinized starfish (DPSF), and deproteinized plus demineralized starfish (DPDMSF), which were used for the preparation of hydrolysates.

#### 3.3.2. Enzymatic Hydrolysis

Three enzymes, namely Food Pro PNL, Corolase 7089, and Corolase 8000 (enzyme activity 100 U/mg), were used for the preparation of hydrolysates from the three starfish samples. First, 15 g of the SF, DPSF, and DPDMSF was taken in 50 mL falcon tubes, separately, and mixed with 0.1 N phosphate buffer, pH 7.0 (1:1 *w/v* ratio). Then, the samples were preheated in a shaking incubator at 55 °C for 10 min. The preheated samples were added with each enzyme at 2% (*w*/*w*), and the hydrolysis was continued for 2 h. After the hydrolysis, the samples were centrifuged at 8500× *g* for 20 min to separate the undissolved matter. Then, the recovered supernatants containing the hydrolysates were heated at 90 °C for 10 min to inactivate the enzymes. Enzyme inactivation was carried out after separating the undissolved matter to avoid leaching of minerals into supernatant, especially in SF and DPSF samples. Later, the hydrolysates were lyophilized into a dry powder.

### 3.4. Determination of Protein Degree of Hydrolysis (DH)

DH was determined on hydrolysate samples (0.5 mL) taken at 0, 20, 40, 60, 80, 100 and 120 min of hydrolysis according to Nielsen, et al. [37] with slight modifications. The hydrolysate sample taken at each time interval was mixed with 3.75 mL o-phthaldialdehyde (OPA) reagent, and vortexed for 5 s, followed by incubation for 2 min at room temperature. Prior to incubation, hydrolysate samples were diluted with pure distilled water to keep the absorbance reading below 1.0, and the absorbance was measured at 340 nm using a spectrophotometer (Cary 60 UV–vis, Agilent technologies, Santa Clara, CA, USA). Serine was used as a standard and the DH was calculated using the following formula:

DH (%) = h/htot × 100

where h = number of peptide bonds cleaved, htot = total number of peptide bonds in the protein.

### 3.5. Characterization of Hydrolysates

#### 3.5.1. Weight Yield and Crude Protein Content

The weight yield of hydrolysates was calculated using the following formula:

Yield (%) = (Weight of freeze-dried hydrolysates)/(Weight of initial wet raw material *) × 100

* Wet raw material refers to the whole starfish in the first treatment, deproteinized starfish in the second treatment, and deproteinized plus demineralized starfish in the third treatment.

The total nitrogen content was determined using a nitrogen analyzer (LECO) following the Dumas principle, and then the crude protein content in the sample was calculated using a nitrogen-to-protein conversion factor of 5.58 [38].

#### 3.5.2. Amino Acid Composition of Hydrolysates

The amino acid composition of the hydrolysates was analyzed based on the method of Ozcan and Senyuva [39] with some modifications. Freeze-dried hydrolysate samples (10 mg) were mixed with 4 mL of 6 N HCl and hydrolyzed at 110 °C for 24 h. Hydrolyzed samples were diluted using 0.2 M acetic acid, and automatically injected to LC/MS (Agilent 1100 HPLC, Waldbron, Germany) in replicates, then compared against amino acid standards. Tryptophan and cysteine were not recovered with this method.

#### 3.5.3. Size Exclusion Chromatography (SEC)

The starfish hydrolysates were subjected to size exclusion chromatography (SEC) to estimate their molecular mass distribution. Briefly, samples were dissolved in MilliQ water to a concentration of 10 mg/mL, centrifuged at 10,000× *g* for 10 min, and then the resulting supernatant was filtered (0.45 µm, Fisher Scientific, Waltham, MA, USA) and used for the SEC analysis. AdvanceBio SEC 150 Å Protein Standard (Agilent Technologies) was used for the study of molecular weights. For each sample, 20 μL was injected into two Agilent Bio SEC columns (150 Å and 100 Å) maintained at a constant temperature of 25 °C. The mobile phase consisted of 30% acetonitrile and 0.05% trifluoroacetic acid in Milli-Q water (*v*/*v*). The chromatographic runs were controlled from the Chromeleon software version 7.2 SR 4 (Thermo Scientific, Waltham, MA, USA). From the chromatographic runs of both the standards and hydrolysates, a UV trace of 214 nm was monitored. The retention times of molecular mass standards were used to calculate the molecular mass distribution of the peptides from the different hydrolysates according to the retention time of the different peaks present in the chromatogram.

#### 3.5.4. ABTS Radical Scavenging Activity Assay

The scavenging activity of the ABTS radical was determined according to the method provided by Palma Esposito et al. [40]. Briefly, an ABTS stock solution (7 mM ABTS, in water) was mixed with 2.45 mM K_2_S_2_O_8_ (final concentration) and left in the dark for 16–18 h at room temperature, allowing ABTS radical cation generation. Then, it was diluted with water to acquire an ABTS working solution whose absorbance is 0.7 ± 0.05 at 734 nm. Moreover, 4 μL (50 mg/mL) of each sample was added to 196 μL of ABTS• + solution, and a 2-fold serial dilution was performed for all the hydrolysates. Samples were kept in the dark at 25–27 °C for 7 min; the absorbance was read at 734 nm. The scavenging activity was expressed using the equation,

Scavenging activity (%) = [(A_control_ − A_sample_)/A_control_] × 100

where A_control_ is the absorbance of the ABTS• + solution, and A_sample_ is the absorbance of the sample mixed with ABTS. Trolox was used as the standard, and the results were expressed in nmol of Trolox equivalent per g of dry weight.

#### 3.5.5. Determination of Cell Density by Trypan Blue Dye Assay

In a microtube, 1 part of 0.4% trypan blue and 1 part of cell suspension (human melanoma cells or fibroblasts) were mixed. The mixture was loaded into a Bürker chamber and, within 5 min of mixing, the dead cells and the total number of cells were counted to evaluate the percentage of viable cells [41].

#### 3.5.6. In Vitro Antiproliferative Assay

Human cells were bought from ATCC (American Type Culture Collection, Manassas, VA, USA). A human melanoma cell (A2058; ATCC^®^ CRL-11147™) and human normal fibroblast (MRC-5; ATCC^®^-CCL-171TM) were cultured in DMEM high-glucose supplemented with 10% fetal bovine serum, 1% L-glutamine, and 1% Pen-Strep solution in a humidified incubator at 37 °C and 5% CO_2_. To estimate the in vitro antiproliferative effects, MRC-5 and A2058 cells were seeded on a 96-well microtiter plate at a density of 1 × 10^4^ cells/well and incubated at 37 °C to allow for cell adhesion on the plates. After 24 h, the medium was replaced with fresh medium containing increasing concentrations of the samples (10 µg/mL, 50 µg/mL, and 100 µg/mL) dissolved in sterilized water (milliQ), and further incubated for 72 h. Each concentration was tested at least in triplicate. After 72 h of treatment, cell viability was assessed using the MTT test 3-(4,5-dimethyl-2-thizolyl)-2,5-diphenyl-2H-tetrazolium bromide (A2231,0001, Applichem Panreac Tischkalender, GmbH, Darmstadt, Germany) [42,43]. MTT/PBS solution (0.5 mg/mL) was then added to the wells and incubated for 3 h at 37 °C in a humidified atmosphere. The reaction was stopped by removal of the supernatant, and the formazan products were dissolved with 100 µL of isopropanol. Absorbance was measured at OD = 570 nm using a microplate reader (MultiskanTM FC Microplate Photometer, Thermo Fisher Scientific, Waltham, MA, USA). The assay was performed according to the manufacturer’s instructions. Cell survival was expressed as a percentage of viable cells in the presence of the tested samples, with respect to untreated control cultures. Extracts with cell viabilities above 50% were not considered active. The percentage of cell viability was calculated as follows: mean (A570–A630) and compared to cells supplemented with media alone. Mean, standard deviation, and statistical analysis were calculated on biological triplicates using GraphPad Prism8 software (GraphPad, San Diego, CA, USA). Considering that this assay was set up as a preliminary screening, no positive control was included, in line with the approach reported by Ghasemi et al., 2021 [44], who highlight that the absence of a positive control is acceptable in an exploratory context.

### 3.6. Statistical Analysis

The preparation of hydrolysates from all the samples was carried out at least twice. Analyses of the hydrolysates were then performed in duplicate, and average values from these analyses were subjected to analysis of variance (ANOVA) to determine significant differences between different hydrolysates. A comparison of means was carried out by Duncan’s multiple range tests [45]. Data was regarded as significantly different when *p* < 0.05. Statistical analysis was performed using the Statistical Package for Social Science (IBM SPSS 28.0 for Windows, SPSS Inc., Chicago, IL, USA).

## 4. Conclusions

Protein hydrolysates were successfully prepared from whole starfish (SF), deproteinized starfish (DPSF), as well as deproteinized and demineralized starfish (DPDMSF) using three enzymes, Food Pro PNL (FP), Corolase 8000 (C8), and Corolase 7089 (C7). The weight yield was highest for the hydrolysates prepared from whole starfish using Food Pro PNL and Corolase 7089, and the hydrolysate produced from deproteinized plus demineralized starfish using Corolase 8000. The hydrolysis of only deproteinized biomass was challenging, resulting in both a low weight yield and low DH, independent of the enzyme type. However, their purity in terms of crude protein content was higher than those prepared from whole starfish. Colorase 8000 was mainly effective on deproteinized and demineralized biomass, resulting in a good weight yield, purity, and DH, indicating specificity in the collagen protein. Though the additional steps added time and cost, they led to purer products with unique bioactivities. The ABTS radical scavenging activity appeared to be weakly influenced by the type of enzyme used, except in the case of whole starfish. Interestingly, the complete absence of minerals due to a demineralization step negatively impacts this biological activity. On the contrary, hydrolysates obtained in the absence of deproteinization and demineralization processes showed antiproliferative activity against melanoma cells, probably ascribed to the presence of minerals and non-collagenous peptides. Overall, the findings underscore the critical importance of carefully selecting the biomass pretreatment method and enzyme type to tailor the weight yield, peptide size, purity, and bioactivity of starfish protein hydrolysates for specific functional and bioactive applications.

## Figures and Tables

**Table 1:** Amino acid composition of whole starfish (SF), deproteinized starfish (DPSF), and demineralized starfish (DPDMSF) hydrolysates, obtained by Food Pro PNL (FP), Corolase 8000 (C8), or Corolase 7089 (C7) (mg/g protein).

Amin Acid/Sample	SF-FP	SF-C8	SF-C7	DPSF-FP	DPSF-C8	DPSF-C7	DPDMSF-FP	DPDMSF-C8	DPDMSF-C7
Lysine	49.48 ± 3.96	44.43 ± 3.31	53.59 ± 1.72	23.28 ± 1.64	46.94 ± 7.92	35.98 ± 2.86	24.03 ± 1.54	47.15 ± 8.39	19.73 ± 0.44
Histidine	4.88 ± 0.67	3.05 ± 0.11	5.21 ± 0.38	2.42 ± 1.24	4.82 ± 1.63	2.73 ± 0.59	0.54 ± 0.13	4.08 ± 1.50	0.02 ± 0.001
Arginine	21.68 ± 1.45	13.53 ± 0.53	23.49 ± 1.44	54.05 ± 16.79	75.61 ± 13.08	57.23 ± 4.11	37.99 ± 1.94	76.02 ± 16.94	23.57 ± 1.31
Glycine	89.29 ± 4.37	75.48 ± 4.57	88.59 ± 4.28	234.46 ± 67.85	326.27 ± 61.86	261.68 ± 21.49	177.53 ± 7.84	305.22 ± 66.62	114.97 ± 8.72
Serine	31.80 ± 1.96	22.31 ± 1.12	31.93 ± 1.72	75.69 ± 23.81	112.93 ± 25.38	88.22 ± 9.55	59.47 ± 2.60	108.71 ± 26.09	42.48 ± 2.25
Alanine	6.56 ± 0.13	5.40 ± 0.18	6.69 ± 0.28	13.14 ± 3.15	17.76 ± 3.04	14.47 ± 0.74	10.70 ± 0.26	17.14 ± 3.82	8.02 ± 0.39
Threonine	1.23 ± 0.32	0.75 ± 0.12	1.21 ± 0.16	1.40 ± 0.13	2.96 ± 0.18	1.56 ± 0.50	0.28 ± 0.08	2.08 ± 0.99	0.15 ± 0.01
Glutamine	70.50 ± 4.13	55.46 ± 3.12	76.23 ± 3.68	129.36 ± 40.26	189.58 ± 37.46	149.98 ± 11.16	108.31 ± 5.33	193.90 ± 40.54	77.03 ± 2.32
Asparagine	54.39 ± 2.65	41.09 ± 2.42	56.95 ± 1.69	86.62 ± 27.15	131.59 ± 28.38	101.08 ± 9.43	69.75 ± 2.91	131.40 ± 33.33	51.53 ± 0.87
Proline	30.61 ± 0.86	18.76 ± 1.09	30.64 ± 1.53	102.98 ± 29.80	149.03 ± 32.169	120.03 ± 7.08	86.87 ± 4.26	147.10 ± 33.78	59.94 ± 3.22
Valine	28.51 ± 1.49	23.24 ± 1.02	29.93 ± 1.83	31.86 ± 9.77	46.67 ± 9.79	36.14 ± 3.12	25.56 ± 1.49	43.96 ± 9.85	20.40 ± 0.12
Methionine	9.61 ± 1.30	5.87 ± 1.42	10.85 ± 0.96	15.78 ± 3.03	27.54 ± 7.41	19.99 ± 2.52	10.59 ± 3.73	25.06 ± 6.36	5.31 ± 1.68
Tyrosine	17.57 ± 2.56	12.41 ± 0.78	17.23 ± 0.54	16.60 ± 5.61	25.64 ± 6.25	16.87 ± 3.67	8.83 ± 0.57	19.06 ± 4.63	7.07 ± 0.20
Isoleucine	23.97 ± 2.02	19.74 ± 0.99	24.88 ± 1.04	25.62 ± 7.45	37.67 ± 7.76	29.71 ± 2.31	21.75 ± 1.17	35.91 ± 8.03	17.37 ± 0.07
Leucine	35.61 ± 2.68	28.95 ± 1.50	37.11 ± 1.37	28.68 ± 5.43	42.90 ± 8.69	33.79 ± 2.63	24.09 ± 1.63	41.67 ± 9.55	20.06 ± 0.93
Phenylalanine	19.64 ± 2.60	15.44 ± 0.90	19.72 ± 1.05	8.66 ± 1.56	15.80 ± 4.05	10.71 ± 0.93	6.12 ± 0.54	13.59 ± 3.92	5.64 ± 0.45

Note: Results are given as mean ± S.D (n = 3).

## Data Availability

Data can be shared if requested.

## References

[1] Clare D.A., Swaisgood H.E. (2000). Bioactive Milk Peptides: A Prospectus. J. Dairy Sci..

[2] Elias R.J., Kellerby S.S., Decker E.A. (2008). Antioxidant Activity of Proteins and Peptides. Crit. Rev. Food Sci. Nutr..

[3] Shahidi F., Zhong Y. (2008). Bioactive Peptides. J. AOAC Int..

[4] De Domenico S., De Rinaldis G., Paulmery M., Piraino S., Leone A. (2019). Barrel Jellyfish (*Rhizostoma pulmo*) as Source of Antioxidant Peptides. Mar. Drugs.

[5] Leone A., Lecci R.M., Durante M., Meli F., Piraino S. (2015). The Bright Side of Gelatinous Blooms: Nutraceutical Value and Antioxidant Properties of Three Mediterranean Jellyfish (Scyphozoa). Mar. Drugs.

[6] Vishkaei M.S., Ebrahimpour A., Abdul-Hamid A., Ismail A., Saari N. (2016). Angiotensin-I Converting Enzyme (ACE) Inhibitory and Anti-Hypertensive Effect of Protein Hydrolysate from *Actinopyga lecanora* (Sea Cucumber) in Rats. Mar. Drugs.

[7] Jo H.Y., Jung W.K., Kim S.K. (2008). Purification and Characterization of a Novel Anticoagulant Peptide from Marine Echiuroid Worm, *Urechis unicinctus*. Process Biochem..

[8] Jung W.K., Kim S.K. (2009). Isolation and Characterisation of an Anticoagulant Oligopeptide from Blue Mussel, *Mytilus edulis*. Food Chem..

[9] Jung W.K., Jo H.Y., Qian Z.J., Jeong Y.J., Park S.G., Choi I.W., Kim S.K. (2007). A Novel Anticoagulant Protein with High Affinity to Blood Coagulation Factor Va from *Tegillarca granosa*. BMB Rep..

[10] Safari R., Yaghoubzadeh Z. (2020). Antioxidant Activity of Bioactive Peptides Extracted from Sea Cucumber (*Holothuria leucospilata*). Int. J. Pept. Res. Ther..

[11] Librizzi M., Martino C., Mauro M., Abruscato G., Arizza V., Vazzana M., Luparello C. (2024). Natural Anticancer Peptides from Marine Animal Species: Evidence from In Vitro Cell Model Systems. Cancers.

[12] Han S.B., Won B., Yang S.C., Kim D.H. (2021). *Asterias pectinifera*-Derived Collagen Peptide-Encapsulating Elastic Nanoliposomes for the Cosmetic Application. J. Ind. Eng. Chem..

[13] Magnesen T., Redmond K.J. (2012). Potential Predation Rates by the Sea Stars *Asterias rubens* and *Marthasterias glacialis*, on Juvenile Scallops, *Pecten maximus*, Ready for Sea Ranching. Aquac. Int..

[14] Vate N.K., Undeland I., Abdollahi M. (2022). Resource Efficient Collagen Extraction from Common Starfish with the Aid of High Shear Mechanical Homogenization and Ultrasound. Food Chem..

[15] Lian K., Maribu I., Rode T.M., Jenssen M., Vang B., Solstad R.G. (2024). More Sustainable Use of Aquaculture Cleaner Fish: Collagen-Rich Protein Hydrolysates from Lumpfish (*Cyclopterus lumpus*)—Effects of Biomass, Pretreatment, and Enzyme Choice. Front. Sustain. Food Syst..

[16] Blowes L.M., Egertová M., Liu Y., Davis G.R., Terrill N.J., Gupta H.S., Elphick M.R. (2017). Body Wall Structure in the Starfish *Asterias rubens*. J. Anat..

[17] Hou N.T., Chen B.H. (2023). Extraction, Purification and Characterization of Collagen Peptide Prepared from Skin Hydrolysate of Sturgeon Fish. Food Qual. Saf..

[18] Nurilmala M., Pertiwi R.M., Nurhayati T., Fauzi S., Batubara I., Ochiai Y. (2019). Characterization of Collagen and Its Hydrolysate from Yellowfin Tuna *Thunnus albacares* Skin and Their Potencies as Antioxidant and Antiglycation Agents. Fish. Sci..

[19] See S.F., Hoo L.L., Babji A.S. (2011). Optimization of Enzymatic Hydrolysis of Salmon (*Salmo salar*) Skin by Alcalase. Int. Food Res. J..

[20] Šližytė R., Daukšas E., Falch E., Storrø I., Rustad T. (2005). Characteristics of Protein Fractions Generated from Hydrolysed Cod (*Gadus morhua*) By-Products. Process Biochem..

[21] Mamelona J., Saint-Louis R., Pelletier É. (2010). Nutritional Composition and Antioxidant Properties of Protein Hydrolysates Prepared from Echinoderm Byproducts. Int. J. Food Sci. Technol..

[22] Chung L., Dinakarpandian D., Yoshida N., Lauer-Fields J.L., Fields G.B., Visse R., Nagase H. (2004). Collagenase Unwinds Triple-Helical Collagen Prior to Peptide Bond Hydrolysis. EMBO J..

[23] Yang Z., Liao Y., Fu X., Zaporski J., Peters S., Jamison M., Liu Y., Wullschleger S.D., Graham D.E., Gu B. (2019). Temperature Sensitivity of Mineral-Enzyme Interactions on the Hydrolysis of Cellobiose and Indican by β-Glucosidase. Sci. Total Environ..

[24] Kittiphattanabawon P., Benjakul S., Visessanguan W., Nagai T., Tanaka M. (2005). Characterisation of Acid-Soluble Collagen from Skin and Bone of Bigeye Snapper (*Priacanthus tayenus*). Food Chem..

[25] Luo P., Hu C.Q., Xia J.J., Ren C.H., Jiang X. (2011). Chemical Constituent Analysis of the Crown-of-Thorns Starfish *Acanthaster planci* and Potential Utilization Value of the Starfish as Feed Ingredient for Animals. Afr. J. Biotechnol..

[26] Abdollahi M., Rezaei M., Jafarpour A., Undeland I. (2018). Sequential Extraction of Gel-Forming Proteins, Collagen and Collagen Hydrolysate from Gutted Silver Carp (*Hypophthalmichthys molitrix*), a Biorefinery Approach. Foods.

[27] Tan C.C., Karim A.A., Latiff A.A., Gan C.Y., Ghazali F.C. (2013). Extraction and Characterization of Pepsin-Solubilized Collagen from the Body Wall of Crown-of-Thorns Starfish (*Acanthaster planci*). Int. Food Res. J..

[28] Lee K.-J., Park H.Y., Kim Y.K., Park J.I., Yoon H.D. (2009). Biochemical Characterization of Collagen from the Starfish *Asterias amurensis*. J. Korean Soc. Appl. Biol. Chem..

[29] Nagai T., Suzuki N. (2000). Partial Characterization of Collagen from Purple Sea Urchin (*Anthocidaris crassispina*) Test. Int. J. Food Sci. Technol..

[30] Munteanu I.G., Apetrei C. (2021). Analytical Methods Used in Determining Antioxidant Activity: A Review. Int. J. Mol. Sci..

[31] Aubry L., Sy K., Sayd T., Ferraro V. (2023). Collagen Peptides-Minerals Complexes from the Bovine Bone By-Product to Prevent Lipids Peroxidation in Meat and Butter and to Quench Free Radicals—Influence of Proteases and of Steam Sterilisation. Appl. Sci..

[32] Elavarasan K., Naveen Kumar V., Shamasundar B.A. (2014). Antioxidant and Functional Properties of Fish Protein Hydrolysates from Fresh Water Carp (*Catla catla*) as Influenced by the Nature of Enzyme. J. Food Process. Preserv..

[33] Kalafatovic D., Giralt E. (2017). Cell-Penetrating Peptides: Design Strategies beyond Primary Structure and Amphipathicity. Molecules.

[34] Lee C.C., Hsieh H.J., Hsieh C.H., Hwang D.F. (2014). Spine Venom of Crown-of-Thorns Starfish (*Acanthaster planci*) Induces Antiproliferation and Apoptosis of Human Melanoma Cells (A375.S2). Toxicon.

[35] Malyarenko T.V., Malyarenko O.S., Kicha A.A., Kalinovsky A.I., Dmitrenok P.S., Ivanchina N.V. (2022). In Vitro Anticancer and Cancer-Preventive Activity of New Triterpene Glycosides from the Far Eastern Starfish *Solaster pacificus*. Mar. Drugs.

[36] Lazzara V., Arizza V., Luparello C., Mauro M., Vazzana M. (2019). Bright Spots in the Darkness of Cancer: A Review of Starfishes-Derived Compounds and Their Anti-Tumor Action. Mar. Drugs.

[37] Nielsen P.M., Petersen D., Dambmann C. (2001). Improved method for determining food protein degree of hydrolysis. J. Food Sci..

[38] Mariotti F., Tomé D., Mirand P.P. (2008). Converting Nitrogen into Protein—Beyond 6.25 and Jones’ Factors. Crit. Rev. Food Sci. Nutr..

[39] Ozcan S., Şenyuva H.Z. (2006). Improved and simplified liquid chromatography/atmospheric pressure chemical ionization mass spectrometry method for the analysis of underivatized free amino acids in various foods. J. Chromatogr. A.

[40] Palma Esposito F., López-Mobilia A., Tangherlini M., Casella V., Coppola A., Varola G., Vitale L., Della Sala G., Tedesco P., Montano S. (2025). Novel Insights and Genomic Characterization of Coral-Associated Microorganisms from Maldives Displaying Antimicrobial, Antioxidant, and UV-Protectant Activities. Biology.

[41] Strober W. (2015). Trypan blue exclusion test of cell viability. Curr. Protoc. Immunol..

[42] Baudelet P.H., Gagez A.L., Bérard J.B., Juin C., Bridiau N., Kaas R., Thiéry V., Cadoret J.-P., Picot L. (2013). Antiproliferative activity of *Cyanophora paradoxa* pigments in melanoma, breast and lung cancer cells. Mar. Drugs.

[43] Martínez K.A., Saide A., Crespo G., Martín J., Romano G., Reyes F., Ianora A. (2022). Promising antiproliferative compound from the green microalga *Dunaliella tertiolecta* against human cancer cells. Front. Mar. Sci..

[44] Ghasemi M., Turnbull T., Sebastian S., Kempson I. (2021). The MTT Assay: Utility, Limitations, Pitfalls, and Interpretation in Bulk and Single-Cell Analysis. Int. J. Mol. Sci..

[45] Steel R.G.D., Torrie J.H. (1980). Principles and Procedures of Statistics: A Biometrical Approach.

